# Incidence of post-intensive care syndrome and its impact on the quality of life of the family caregiver

**DOI:** 10.15649/cuidarte.4527

**Published:** 2025-08-13

**Authors:** Marcia Andrea Quiñonez - Mora, Johan Sebastián Villada - Gómez, Hoover León - Giraldo, Alexander Casallas - Vega

**Affiliations:** 1 Los Cobos Medical Center, Bogotá, Colombia. E-mail: andreaqenf@yahoo.es Los Cobos Medical Center Bogotá Colombia andreaqenf@yahoo.es; 2 Universidad de Caldas, Bogotá, Colombia. E-mail: johan.villada@ucaldas.edu.co Universidad de Caldas Bogotá Colombia johan.villada@ucaldas.edu.co; 3 Universidad Libre, Bogotá, Colombia. E-mail: hooverle25@gmail.com Universidad Libre Bogotá Colombia hooverle25@gmail.com; 4 Fundación Universitaria de ciencias de la Salud, Bogotá, Colombia. E-mail: acasallas1@fucsalud.edu.co Fundación Universitaria de ciencias de la Salud Bogotá Colombia acasallas1@fucsalud.edu.co

**Keywords:** Critical Care, Critical Care Outcomes, Family, Anxiety, Depression, Quality of Life, Cuidados Críticos, Resultados de Cuidados Críticos, Familia, Ansiedad, Depresión, Calidad de Vida, Cuidados Críticos, Resultados de Cuidados Críticos, Família, Ansiedade, Depressão, Qualidade de Vida

## Abstract

**Introduction:**

The American Association of Critical Care defines Post-Intensive Care Syndrome (PICS) as a set of new or worsening impairments in the physical, cognitive, or mental health of patients as a result of their stay in the Intensive Care Unit (ICU). Families and caregivers may also experience a form of PICS, referred to as PICS-family, which includes symptoms such as depression, anxiety, post-traumatic stress disorder, and sleep disturbances.

**Objective:**

To determine the incidence of PICS-F in a high-complexity healthcare facility and its relationship with the quality of life of family caregivers.

**Materials and Methods:**

A prospective, analytical, longitudinal, and observational study was conducted with 95 family caregivers of patients admitted to the intensive care unit (ICU).

**Results:**

An incidence of 9.4 and 11.6 cases of PICS-F per 100 ICU days was identified. This incidence tends to decrease over time. A statistically significant association was found between the presence of anxiety and the development of depression, with an OR of 5.49 [95% IC: 2-14] (p=0.001). Family caregivers reported a negative perception of their quality of life across all three measurement points.

**Discussion:**

Anxiety was found to affect all four dimensions of quality of life negatively. The results may be associated with the high levels of stress experienced during the initial stages of ICU admission.

**Conclusion:**

PICS-F was identified as a condition that affects family caregivers with anxiety and depression, adversely affecting all four dimensions of the caregiver's quality of life.

## Resumen

**Introducción:**

La Asociación Americana de Cuidado Crítico define el síndrome post UCI (PICS) como el conjunto de alteraciones nuevas que empeoran la salud física, cognitiva o mental de los pacientes como consecuencia de la estancia en la Unidad de Cuidados Intensivos. Las familias y los cuidadores también pueden sufrir una forma de PICS denominada PICS-familia, que incluye la presencia de depresión, ansiedad, trastorno de estrés postraumático y alteraciones de sueño.

**Objetivo:**

Determinar la incidencia del PICS – F en una institución de alta complejidad y su relación con la calidad de vida del cuidador familiar.

**Materiales y métodos:**

Estudio observacional analítico, longitudinal, prospectivo que incluyó 95 cuidadores familiares de pacientes que ingresaron a UCI.

**Resultados:**

Se identificó una incidencia de PICS – F de 9,4 casos y de 11, 6 casos por cada 100 días de cuidado en el entorno del cuidado crítico, esta incidencia tiende a disminuir en el paso del tiempo, se encontró una asociación estadísticamente significativa entre la presencia de ansiedad y el desarrollo de depresión con un OR 5,49 [2-14] (p=0,001). Los cuidadores familiares perciben su calidad de vida de manera negativa en los tres momentos de la medición.

**Discusión:**

La presencia de ansiedad afecta las cuatro dimensiones de la calidad de vida, estos resultados pueden estar relacionados con los altos niveles de estrés que se vivencian al momento de ingreso del paciente.

**Conclusiones:**

El PICS-F se identificó como una condición que afecta los cuidadores familiares dado que la ansiedad y la depresión impactan negativamente las cuatro dimensiones de calidad de vida del cuidador.

## Resumo

**Introdução:**

A Associação Americana de Unidades de Terapia Intensiva (UTI) define a síndrome pós-UTI (PICS) como o conjunto de novas alterações que pioram a saúde física, cognitiva ou mental dos pacientes em consequência de sua permanência na Unidade de Terapia Intensiva. Famílias e cuidadores também podem apresentar uma forma de PICS chamada PICS-familiar, que inclui depressão, ansiedade, transtorno de estresse pós-traumático e distúrbios do sono.

**Objetivo:**

Determinar a incidência de PICS – F em uma instituição de alta complexidade e sua relação com a qualidade de vida do cuidador familiar.

**Materiais e métodos:**

Estudo observacional analítico, longitudinal, prospectivo, que incluiu 95 cuidadores familiares de pacientes internados em UTI.

**Resultados:**

Foi identificada uma incidência de PICS – F de 9,4 casos e 11,6 casos por 100 dias de atendimento no ambiente de terapia intensiva, essa incidência tende a diminuir ao longo do tempo, foi encontrada associação estatisticamente significativa entre a presença de ansiedade e o desenvolvimento de depressão com OR 5,49 [2-14] (p=0,001). Os cuidadores familiares percebem sua qualidade de vida negativamente em todos os três pontos de medição.

**Discussão:**

A presença de ansiedade afeta as quatro dimensões da qualidade de vida, resultados que podem estar relacionados aos altos níveis de estresse vivenciados no momento da admissão do paciente.

**Conclusões:**

O PICS-F foi identificado como uma condição que afeta os cuidadores familiares, pois a ansiedade e a depressão impactam negativamente todas as quatro dimensões da qualidade de vida do cuidador.

## Introduction

The American Association of Critical Care defines post-intensive care syndrome (PICS) as a set of new or worsening impairments in the physical, cognitive, or mental health status of patients resulting from their stay in the Intensive Care Unit (ICU)^1^. 

PICS-Family (PICS-F)^2^,^3^ refers to the psychological symptoms experienced by family caregivers of ICU patients, which often persist beyond the patient's discharge from the ICU. These symptoms include depression, anxiety, post-traumatic stress disorder (PTSD), and sleep disturbances^2^–^4^. Their incidence ranges from 30% to 75%^5^,^6^. Several authors have reported depressive symptoms in 25% to 90% of informal caregivers^4^,^7^, anxiety in 20% to 80%^3^,^7^, and PTSD in 30%^3^,^7^. Depression is the most commonly experienced symptom, followed by anxiety^8^. These symptoms have been identified as early as days 3 to 5 after ICU admission^9^ and may persist for up to 4 years after discharge^10^.

Different risk factors contribute to the development of PICS-F. Caregiver-related factors include being female, caring for a spouse, having a low educational level, pre-existing mental or physical health conditions, lack of social support, and a caregiving burden greater than 100 hours per month. Patient-related factors include the severity of illness, reduced mobility, and dependence for basic activities. Health system-related factors include lack of health insurance and the absence of professional home care. Risk factors during ICU hospitalization include restricted visiting hours, caregivers’ perception of impending death, and poor communication with medical staff^3^. These symptoms are often unrecognized by healthcare professionals, resulting in a lack of necessary support for caregivers^8^.

Ferrell, as cited by Vega-Angarita et al.^11^, states that quality of life refers to "the positive or negative attributes that characterize the life of the family caregiver on four dimensions: physical, psychological, spiritual, and social well-being."

Evidence shows that the quality of life of family caregivers is inversely related to the presence of PICS-F^10^. Inadequate management of these symptoms may result in mistreatment, abuse, or neglect of both the survivor and the family^7^. Caregivers' compromised mental health can hinder their ability to support ICU survivors during recovery^7^. This creates a cycle in which both survivors and their family caregivers are affected emotionally, physically, and psychologically. The objective of this study was to determine the incidence of PICS-F and its relationship with the quality of life of family caregivers in a high-complexity healthcare facility in Bogotá, Colombia. 

## Materials and Methods

A prospective, analytical, longitudinal, and observational study was conducted among family caregivers of patients admitted to the ICU of a high-complexity healthcare facility in Bogotá, Colombia. This ICU has 24 beds and treats patients with medical and surgical conditions. Approximately 1,800 patients are admitted annually.

The study population consisted of 95 family caregivers of ICU patients admitted between January and April 2023 who voluntarily agreed to participate in the study.

The research team designed a data collection form composed of three sections:

- Sociodemographic data of the patient and family caregiver. For patients, the data included medical diagnosis, need for ventilatory support and its duration, length of ICU stay, illness severity (as measures by the PACHE scale), mobility limitations, and need for assistance with basic activities. For family caregivers, data included gender, relationship to the patient, educational level, place of origin and residence, marital status, occupation, and socioeconomic status.

- Goldberg Anxiety and Depression Scale (GADS), validated in Spanish. This 18-item scale comprises two subscales: the first (items 1-9) assesses anxiety, and the second (items 10-18) assesses depression, both of which are answered with either "yes" (1 point) or "no" (0 points), yielding a total score ranging from 0 to 18. A score of 4 or more on the anxiety subscale indicates the presence of anxiety, while a score of 2 or more on the depression subscale indicates depression. The anxiety subscale has a sensitivity of 82% and a positive predictive value (PPV) of 0.56. The depression subscale has a sensitivity of 85% and a PPV of 0.85. The specificity for both subscales is 91%^12^.

- To measure the quality of life of family caregivers, we used the instrument developed by Ferrell and validated in Spanish by Barrera et al.^13^ The scale consists of 35 items rated on a 4-point Likert scale. It is divided into four dimensions: physical, psychological, social, and spiritual. The physical dimension includes 5 items with a score range from 5 to 20. The psychological dimension consists of 14 items, with a range of 14 to 56. The social dimension has 9 items, ranging from 9 to 36. The spiritual dimension consists of 7 items, with scores ranging from 7 to 28^11^.

- In the physical, social, and spiritual dimensions, lower scores reflect a more positive perception of the caregiver’s health status, social interactions, and spirituality, respectively. In contrast, lower scores in the psychological dimension reflect a more negative perception of emotional well-being. The overall content validity index of the instrument is 0.9, and the Cronbach’s alpha coefficient is 0.84^11^.

The sample size was estimated using the Epidat 4.2 calculator based on a 95% confidence level and a 5% margin of error. According to the ICU’s epidemiological report, 336 patients were discharged between January and April 2022, a figure that was considered to be the target population size. The sample initially consisted of 101 family caregivers. However, six critically ill patients died during the data collection period, resulting in the withdrawal of their family members from the study. Thus, the final sample included 95 family caregivers. The instruments were administered at three time points: upon ICU admission and at 15 and 30 days after ICU admission.

For data analysis, the information was compiled in a Microsoft Excel© database and analyzed using the SPSS version 27 statistical package. Categorical variables were reported as absolute frequencies and percentages, and medians and quartiles were calculated. Quantitative variables were described using measures of central tendency and dispersion, depending on the data distribution, which was assessed using the Kolmogorov–Smirnov test for normality.

Both cumulative incidence (CI) and incidence density (ID) were calculated. Cumulative incidence was compared across the three measurement points using the chi-square test. Given the limited number of events of interest relative to the sample size, estimates were made under the assumption of a Poisson distribution. Both point estimates and 95% confidence intervals were calculated for incidence density. Additionally, the relationship between depression and anxiety was analyzed by estimating odds ratios (OR) at admission and 15 days and 30 days after admission.

A logistic regression analysis was conducted to examine the relationship between the presence of PICS-F and the family caregiver’s quality of life. Odds ratios (OR) with 95% confidence intervals were estimated at each of the three measurement points: at ICU admission and 15 and 30 days post-admission. All collected data are freely accessible and available for consultation on Mendeley Data^14^.

Participation in the study was anonymous and voluntary, with informed consent obtained prior to administering the instruments. The study adhered to the ethical principles outlined in the Declaration of Helsinki, the Nuremberg Code, and the Belmont Report, as well as the provisions of Resolution 8430 of 1993, which establishes the scientific, technical, and administrative standards for health research in Colombia. This study was submitted to and approved by the research committee (CoordInv/103) and the ethics and research committees of the participating institutions (CIE 025-2022) (CBCS-059).

## Results

After analyzing the database of the 95 caregivers, the following results were obtained: 61.10% (58 out of 95) were women, with a mean age of 50.2 years. Most participants were married, and the most frequent family caregivers in this study were children of critically ill patients (44.20%) (Table 1). 

**Table 1:** Sociodemographic characteristics of family caregivers of ICU patient

Sociodemographic and clinical characteristics Description	Summary statistics %(n)
Sex	
Women	61.10 (58)
Men	38.90 (37)
Age (years)	
Mean (SD)	50.19 ± 16.40
Median [IQR]	50 [ 20; 86]
Marital status	
Married	48.40 (46)
Single	23.20 (22)
Cohabiting	22.10 (21)
Separated	4.20 (4)
Widowed	2.10 (2)
Socioeconomic status	
Low	20.00 (19)
Middle	78.90 (75)
High	1.10 (1)
Educational level	
Elementary	7.40 (7)
High School	14.70 (14)
Technical degree	17.90 (17)
Bachelor’s degree	33.70 (32)
Graduate degree	26.30 (25)
Family caregiver occupation	
Employee	41.10 (39)
Self-employed	25.30 (24)
Retired	21.10 (20)
Homemaker	12.60 (12)
Kindship of the family caregiver with the critically ill patient.	
Child	44.20 (42)
Spouse	38.90 (37)
Parent	8.40 (8)
Sibling	5.30 (5)
Other	3.20 (3)
Locality of origin (Bogotá)	
Suba	24.20 (23)
Usaquén	14.70 (14)
Fontibón	5.30 (5)
Kennedy	5.30 (5)
Bosa	3.20 (3)
Engativá	3.20 (3)
Teusaquillo	2.10 (2)
Tunjuelito	2.10 (2)
Antonio Nariño	1.10 (1)
Barrios Unidos	1.10 (1)
Chapinero	1.10 (1)
Ciudad Bolívar	1.10 (1)
Puente Aranda	1.10 (1)
Rafael Uribe	1.10 (1)
San Diego	1.10 (1)
Santafé	1.10 (1)
Usme	1.10 (1)
Origin outside Bogotá	
Cundinamarca	26.30 (25)
Caldas	2.10 (2)
Antioquia	1.10 (1)
Boyacá	1.10 (1)

SD: Standard Deviation. IR: Interquartile range

Regarding the characteristics of the critically ill patients, the majority were female (55.80%), with a mean age of 63 years (SD = 18). The median length of stay in the ICU was 3 days, with an interquartile range (IQR) of 2 to 4 days. The primary reason for ICU admission was a medical diagnosis. By the final measurement point, most patients (75.80%) were classified as independent in performing activities of daily living.

**Figure 1:**
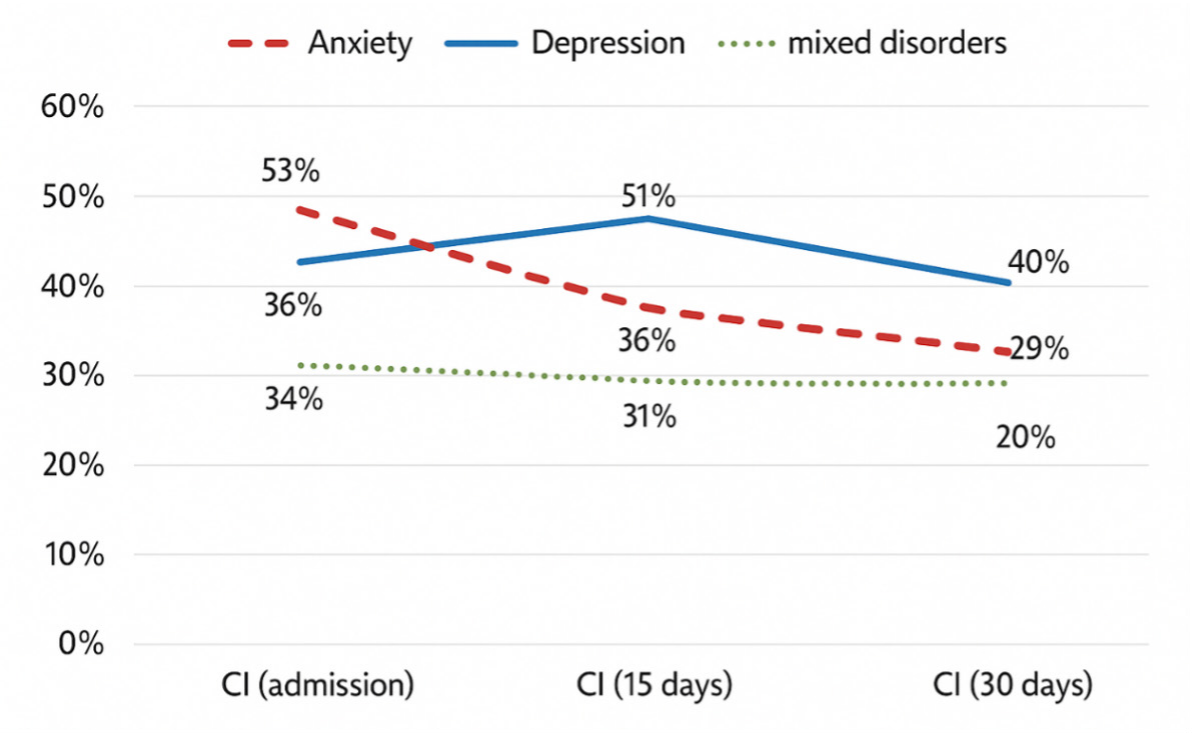
Cumulative incidence of anxiety, depression, and mixed disorders

The incidence rate of PICS-F anxiety/depression (expressed as cases per 100 ICU days) was 9.4 cases for anxiety, 11.6 for depression, and 9.4 for mixed disorders. The cumulative incidence is shown in Figure 1. For anxiety, a statistically significant decrease in cumulative incidence was observed across the three measurement points (p = 0.003). In contrast, the incidence of depression did not show a statistically significant change at the three measurement points. 

When examining the association between anxiety and depression in family caregivers, it was found that at ICU admission, 50 out of 95 participants screened positive for anxiety, and among them, 32 also presented with depression (OR:5.49; 95% CI 2.00 -14.89; p=0.001) (Table 2). 

**Table 2:** Existing association between anxiety and depression in family caregivers of critically ill patients

Factor Anxiety	Depression		OR (CI 95%)	p-value
	Yes	No		
Upon ICU admission	(43)	(52)		
Yes	74.42 (32)	34.62 (18)	1	
No	25.58 (11)	65.38 (34)	5.49 (2.07 – 14.98)	<0.001
15 days post-ICU admission	(48)	(47)		
Yes	60.42 (29)	10.64 (5)	1	
No	39.58 (19)	89.36 (42)	12.80 (3.95- 47.64)	<0.001

OR (95% CI): Odds Ratio (Confidence Interval 95%)

Regarding the quality of life at ICU admission, family caregivers reported a median score of 10 for physical well-being, with a first quartile (Q1) of 8 and a third quartile (Q3) of 13. For psychological well-being, family caregivers reported a median score of 44, with a Q1=40 and a Q3=47. In the social well-being dimension, the median was 21, with a Q1=18 and a Q3=26. For spiritual well-being, the median was 23, with Q1= 21 and Q3=25. 

On day 15 (second measurement point), family caregivers reported a median physical well-being score of 8, with Q1 = 7 and Q3 = 10. For psychological well-being, the median was 47, with Q1 = 45 and Q3 = 48. In the social well-being dimension, the median was 21, with Q1 = 19 and Q3 = 23. For spiritual well-being, the median was 22, with Q1 = 20 and Q3 = 24. 

**Figure 2:**
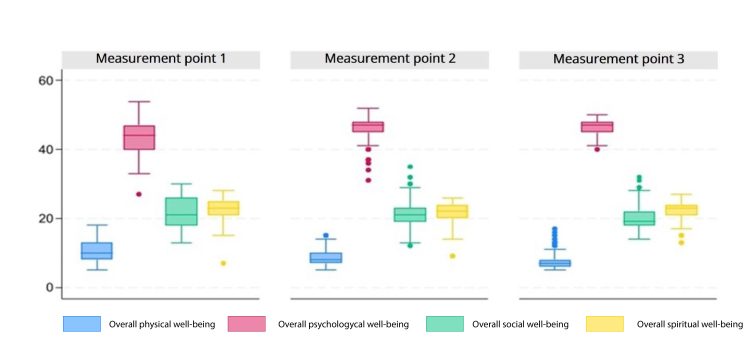
Quality of life of family caregivers

On day 30 (third measurement point), family caregivers had a median score of 7 for physical well-being, with Q1 = 6 and Q3 = 10. For psychological well-being, the median remained at 47, with Q1 = 45 and Q3 = 48. In the social well-being dimension, the median was 19, with Q1 = 18 and Q3 = 22. For spiritual well-being, the median was 23, with Q1 = 21 and Q3 = 24. The quality-of-life scores of family caregivers in the present study are shown in Figure 2. 

*Measurement point 1 (On admission to ICU), Measurement point 2 (15 days), Measurement point 3 (30 days).*

At the first measurement point, the quality of life of family caregivers was interpreted as follows. For physical well-being, 52 participants scored above the median, indicating a negative perception of their health status. In terms of psychological well-being, 51 participants scored above the median, which reflects a positive perception of their emotional dimension. In terms of social well-being, 56 participants scored above the median, indicating a negative perception of their social interactions. For spiritual well-being, 51 participants also scored above the median, suggesting a negative perception of their spiritual dimension.

By the second measurement point, an increase in negative perception of health status was observed. In terms of psychological well-being, 52 participants showed a positive perception of their emotional dimension. Regarding social well-being, 50 participants have a negative perception of their social interactions. For spiritual well-being, 57 of the participants have a negative perception of spirituality.

**Figure 3:**
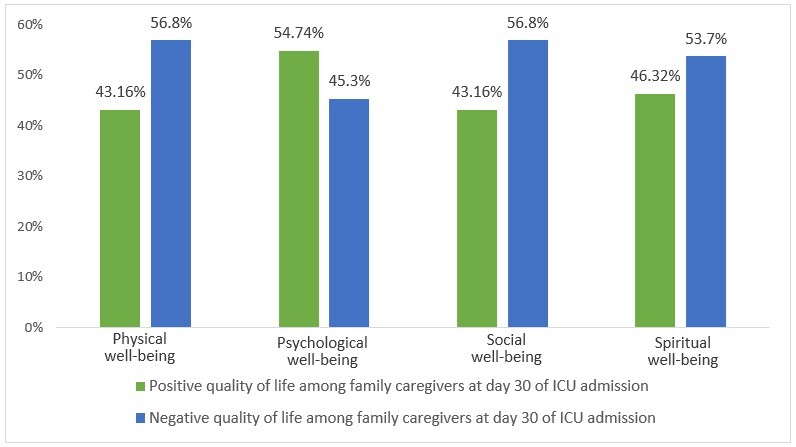
Quality of life of family caregivers at day 30 of ICU admission

At the third measurement point, in terms of physical well-being, 54 participants reported a negative perception of their health status. In terms of psychological well-being, 52 participants have a positive perception of their emotional dimension. Regarding social well-being, 54 participants have a negative perception of their social interactions. For spiritual well-being, 51 participants scored above the median, which also indicates a negative perception of their spirituality (see Figure 3).

A statistically significant association was observed between physical well-being and caregiver anxiety on admission to the ICU, with a 4.7-fold greater probability of a negative assessment of health status in caregivers who were anxious compared to those who were not (p=0.001). This association increased at 15 days (OR = 10) and 30 days (OR = 18.1) (see Table 3). 

**Table 3:** Association between PICS-F (Anxiety) and quality of life of the family caregiver

PICS-F Dimensions	Negative	Positive	OR (CI 95%)	p-value
Physical well-being				
Measurement point 1	52	43		
No Anxiety	30.80 (16)	67.40 (29)	1	
Yes Anxiety	69.20 (36)	32.60 (14)	4.66 (1.96 - 11.10)	<0.001
Measurement point 2	56	39		
No Anxiety	46.40 (26)	89.70 (35)	1	
Yes Anxiety	53.60 (30)	10.30 (4)	10.10 (3.16 - 32.21)	<0.001
Measurement point 3	54	41		
No Anxiety	51.85 (28)	95.10 (39)	1	
Yes Anxiety	48.15 (26)	4.90 (2)	18.11 (3.97 - 82.60)	<0.001
Psychological well-being				
Measurement point 1	44	51		
No Anxiety	68.20 (30)	29.40 (15)	1	
Yes Anxiety	31.80 (14)	70.60 (36)	0.199 (0.08 - 0.466)	<0.001
Measurement point 2	43	52		
No Anxiety	65.12 (28)	63.46 (33)	1	
Yes Anxiety	34.88 (15)	36.54 (19)	0.93 (0.40 - 2.16)	0.867
Measurement point 3	43	52		
No Anxiety	79.07 (34)	63.46 (33)	1	
Yes Anxiety	20.93 (9)	36.54 (19)	0.46 (0.18 - 1.16)	0.100
Social well-being				
Measurement point 1	56	39		
No Anxiety	30.36 (17)	71.79 (28)	1	
Yes Anxiety	69.64 (39)	28.21 (11)	5.84 (2.37 - 14.37)	<0.001
Measurement point 2	50	45		
No Anxiety	40.00 (20)	91.11 (41)	1	
Yes Anxiety	60.00 (30)	8.89 (4)	15.38 (4.76 - 49.65)	<0.001
Measurement point 3	54	41		
No Anxiety	51.85 (28)	95.12 (39)	1	
Yes Anxiety	48.15 (26)	4.88 (2)	18.11 (3.97 - 82.62)	<0.001
Spiritual well-being				
Measurement point 1	53	42		
No Anxiety	56.60 (30)	35.71 (15)	1	
Yes Anxiety	43.40 (23)	64.29 (27)	0.43 (0.185 - 0.98)	0.045
Measurement point 2	57	38		
No Anxiety	63.16 (36)	65.79 (25)	1	
Yes Anxiety	36.84 (21)	34.21 (13)	1.12 (0.48 - 2.65)	0.793
Measurement point 3	51	44		
No Anxiety	64.71 (33)	77.27 (34)	1	
Yes Anxiety	35.29 (18)	22.73 (10)	1.85 (0.75 - 4.60)	0.183

Measurement point 1 (On admission to ICU), Measurement point 2 (15 days), Measurement point 3 (30 days). OR (95% CI): Odds Ratio (Confidence Interval 95%)

A significant association was observed between social well-being and depression in family caregivers upon ICU admission. Caregivers who presented with depression were 3.39 times more likely to have a negative perception of their social well-being compared to those without depression (p = 0.006) (see Table 4). 

**Table 4:** Association between PICS- F (Depression) and quality of life of the family caregiver

PICS-F Dimensions	Negative	Positive	OR (CI 95%)	p-value
Physical well-being				
Measurement point 1	52	43		
No Depression	38.46 (20)	74.42 (32)	1	
Yes Depression	61.54 (32)	25.58 (11)	4.65 (1.92 - 11.27)	<0.001
Measurement point 2	56	39		
No Depression	32.14 (18)	74.36 (29)	1	
Yes Depression	67.86 (38)	25.64 (10)	6.12 (2.46 - 15.23)	<0.001
Measurement point 3	54	41		
No Depression	35.19 (19)	92.68 (38)	1	
Yes Depression	64.81 (35)	7.32 (3)	23.33 (6.35 - 85.70)	<0.001
Psychological well-being				
Measurement point 1	44	51		
No Depression	68.18 (30)	43.14 (22)	1	
Yes Depression	31.82 (14)	56.86 (29)	0.35 (0.152 - 0.82)	0.016
Measurement point 2	43	52		
No Depression	55.81 (24)	44.23 (23)	1	
Yes Depression	44.19 (19)	55.77 (29)	0.63 (0.28 - 1.42)	0.262
Measurement point 3	43	52		
No Depression	67.44 (29)	53.85 (28)	1	
Yes Depression	32.56 (14)	46.15 (24)	0.56 (0.24 - 1.30)	0.180
Social well-being				
Measurement point 1	56	39		
No Depression	42.86 (24)	71.79 (28)	1	
Yes Depression	57.14 (32)	28.21 (11)	3.39 (1.41 - 8.14)	0.006
Measurement point 2	50	45		
No Depression	22.00 (11)	80.00 (36)	1	
Yes Depression	78.00 (39)	20.00 (9)	14.18 (5.27 - 38.19)	<0.001
Measurement point 3	54	41		
No Depression	35.19 (19)	92.68 (38)	1	
Yes Depression	64.81 (35)	7.32 (3)	23.33 (6.35 - 85.73)	<0.001
Spiritual well-being				
Measurement point 1	53	42		
No Depression	62.26 (33)	45.24 (19)	1	
Yes Depression	37.74 (20)	54.76 (23)	0.5 (0.22 - 1.14)	0.100
Measurement point 2	57	38		
No Depression	43.86 (25)	57.89 (22)	1	
Yes Depression	56.14 (32)	42.11 (16)	1.76 (0.77 - 4.04)	0.182
Measurement point 3	51	44		
No Depression	52.94 (27)	68.18 (30)	1	
Yes Depression	47.06 (24)	31.82 (14)	1.90 (0.82 - 4.41)	0.132

Measurement point 1 (On admission to ICU), Measurement point 2 (15 days), Measurement point 3 (30 days). OR (95% CI): Odds Ratio (Confidence Interval 95%)

## Discussion

The cumulative incidence of PICS-F found was 9.4, 11.6, and 9.4 cases per 100 ICU days for anxiety, depression, and mixed disorders, respectively. The literature search revealed no prior studies reporting measurement of PICS-F incidence for comparison, which makes this study novel; however, it also presents a limitation for discussing the results. Nevertheless, Jones et al.^15^ reported in their study a prevalence of anxiety and depression of 61% and 26%, respectively, during the first week after ICU discharge. Similarly, Anderson et al.^16^ found that among relatives of adult ICU patients, 42% exhibited symptoms of anxiety and 16% of depression. In a follow-up of the same group one month after ICU discharge, 21% had anxiety, and 8% had depression^17^. Likewise, Bryant^8^ and Petrinec^10^ described how the prevalence of anxiety tends to decrease over time (45.8% at admission, 34.2% at one-month post-discharge, and 30.6% at three months), whereas depressive symptoms, initially less frequent, tended to increase over time (14.6% at admission, 21.1% at one month, and 25% at three months)^8^.

Most of the participants were women, which is consistent with findings in other studies^7^,^8^ and is related to the historically assumed caregiving role of women. The majority were the children of the critically ill patients, which aligns with findings reported by Petrinec^10^ but differs from Schmidt^9^, who identified spouses as the predominant caregivers of critically ill patients. This may help explain the lower average age of caregivers in the present study.

Most of these patients did not require mechanical ventilation. This result is contrary to the evidence suggesting that approximately 51% of critically ill patients require it^7^ and is a consequence of the relatively short ICU stays and the institutional profile of the facility studied. By day 30 after ICU admission, most patients had high scores on the Barthel Index, indicating independence in daily living activities. This finding suggests that early mobilization and a low prevalence of acquired muscle weakness are consistent with the observed short ICU stays, limited need for mechanical ventilation, and the clinical profile of the patient population.

The results of the present study revealed a statistically significant association between PICS-F anxiety and the family caregiver's quality of life at the first measurement point. This finding suggests that the presence of anxiety negatively impacts all four dimensions of caregiver quality of life.

These results may be explained by the elevated stress experienced when a patient is admitted to the ICU. In this regard, LaBuzetta et al.^18^ note that ICU admission is typically a sudden, devastating, and potentially life-threatening event that causes substantial emotional distress for family caregivers. Effective communication between the healthcare team and caregivers is the cornerstone to mitigate anxiety during ICU admissions. In the present study, the decrease in anxiety evidenced at admission and on day 15 could be attributed to communication, as the nursing staff's education and information in open-door ICUs, as well as the presence of family during ICU medical rounds, are practices that can improve communication and reduce anxiety in family members^3^. Such actions have lasting implications beyond discharge, as adverse outcomes in caregivers can compromise not only their own health but also the recovery and health of the patient^3^.

## Conclusions

The impact of critical illness on family caregivers includes sleep deprivation, fear, and anxiety, symptoms that are exacerbated in ICUs with restricted visitation policies and ineffective communication with healthcare personnel^3^,^17^,^18^. This scenario highlights the importance of caring for the physical and mental health of family caregivers. Those who perceive their health as compromised experience a greater caregiving burden compared to those in good physical condition. This increased burden is associated with a higher prevalence of psychological symptoms and a lower quality of life for caregivers^7^.

As part of the multidimensional and humanized care in the ICU, symptoms of anxiety and depression in family members should also be acknowledged in order to offer psychological support that could have an impact on outcomes.

In caring for the patient-family caregiver dyad, healthcare professionals should ensure that caregivers have access to home care as recommended by medical professionals, prior to discharge (including home oxygen therapy, rehabilitation, and medications. Moreover, caregivers should be trained in basic home care activities for ICU survivors, as well as the safe administration of medications.
